# Necrotizing fasciitis of the neck and head complicated with chronic osteomyelitis: Case report presentation

**DOI:** 10.1016/j.ijscr.2019.03.025

**Published:** 2019-03-26

**Authors:** Rosa María Wong-Chew, Antonio H. Angel-Ambrocio, Sheila Yadira Gomez-Murillo, Angel Puente-Sanchez, Gerardo Fernandez-Sobrino, Alfonso Marhx-Bracho, María de Lourdes Marroquín-Yañez

**Affiliations:** a Facultad de Medicina, Universidad Nacional Autónoma de México, Mexico; b Hospital Angeles del Pedregal, Mexico; c Instituto Nacional de Pediatría, Mexico; d Hospital Infantil de México Federico Gómez, Mexico

**Keywords:** NF, necrotizing fasciitis, VAC, vacuum assisted closure, Necrotizing fasciitis, Children, Osteomyelitis, Multidrug resistant organisms, Case report

## Abstract

**Introduction:**

Necrotizing fasciitis (NF) is a severe infectious condition associated with significant morbidity and mortality and characteristically has a higher incidence in adults; pediatric cases are very rare. In this case report we describe, the clinical presentation, management and outcome of a patient with necrotizing fasciitis of the neck and head complicated with chronic osteomyelitis.

**Presentation of the case:**

An 18 month-old, latin, male, diagnosed with rickets and Crouzon syndrome, twenty-four hours post- craniofacial remodeling surgery presented tissue edema, indurated skin and violaceous and well delimited lesions in the neck and head. The patient developed septic shock, acute osteomyelitis and infection of the cranial grafts. Multiple surgical lavages, debridation of the necrotic tissue and resection of the affected bone, in combination with multiple antibiotics, laboratory test methods, VAC therapy and hyperbaric oxygen were required for successful treatment and full recovery of the patient.

**Discussion:**

Although early antibiotic therapy and critical care are basic for the treatment of patients with NF, early aggressive surgical treatment is crucial for an adequate outcome. Adjuvant treatment for NF such as the Vacuum-assisted closure and the hyperbaric oxygen therapy are very useful to accelerate wound healing. Moreover, special laboratory test methods such as the checkerboard can be used to determine the adequate antimicrobial combination of drugs in the case of multi resistant organisms.

**Conclusions:**

Early diagnosis, adequate antibiotic therapy and aggressive surgical debridement of the necrotic tissue combined with VAC and hyperbaric oxygen therapy played an important role in the successful treatment of NF.

## Highlights

• Necrotizing fasciitis of the head and neck in children is very rare.
• Proper early diagnosis and adequate antibiotic therapy played an important role.
• Early aggressive surgical treatment is crucial for an adequate outcome.
• The vacuum-assisted closure and hyperbaric oxygen therapy are very useful to accelerate wound healing.
• A multidisciplinary management is fundamental to increase treatment success.

## 1 Introduction

Necrotizing fasciitis (NF) is a severe infectious condition associated with significant morbidity and mortality and characteristically has a higher incidence in the adult population [1]. However little is known in pediatric patients [2], its pathogenesis is characterized by bacterial invasion of subcutaneous tissues, rapid horizontal spread of infection along the deep fascial planes, and release of bacterial toxins, which results in tissue ischemia and liquefactive necrosis, as well as fulminant systemic disease including septic shock and death [3].

The Crouzon syndrome is a genetically inherited syndrome characterized by craniosynostosis, resulting in skull and facial deformities detected at birth. The characteristic features are brachycephaly, a flattened forehead, hypertelorism, proptosis, a beaked nose, and maxillary/midface hypoplasia. Surgical management is the treatment of choice to correct the deformity of the midface and orbits to prevent blindness and intellectual disability due to the restriction of the brain and orbital development [4].

We present a case of necrotizing fasciitis in a patient with Crouzon Syndrome, with cranio-cervico-facial involvement after a surgical procedure of craniofacial advancement with septic shock and acute and chronic osteomyelitis with multiple bacteria isolated including multidrug resistant *Pseudomonas aeruginosa*. The diagnosis and management of the patient was perfomed at a private institution.

This work has been reported in line with the SCARE criteria [5].

## 2 Case presentation

An 18 month-old, latin, male, diagnosed with Rickets and Crouzon syndrome, received pharmacological treatment for rickets during three months. He was referred to neurosurgery due to occipital protrusions and skull deformity. A cranial remodeling was performed, the surgery concluded successfully without complications, a surgical drain was placed and antimicrobial prophylaxis (cephalothin) was given. On the first postoperative day, the patient presented fever (38.6 °C) tachycardia, tachypnea and dyspnea. Laboratory results, showed a white cell count of 3.9 × 10^3^/mm^3^ and platelets of 82 × 10^3^/mm^3^. Over the next 48 h, antibiotic therapy was changed to third generation cephalosporin (ceftriaxone) due to the persistence of fever and the presence of diarrhea.

**Fig. 1:**
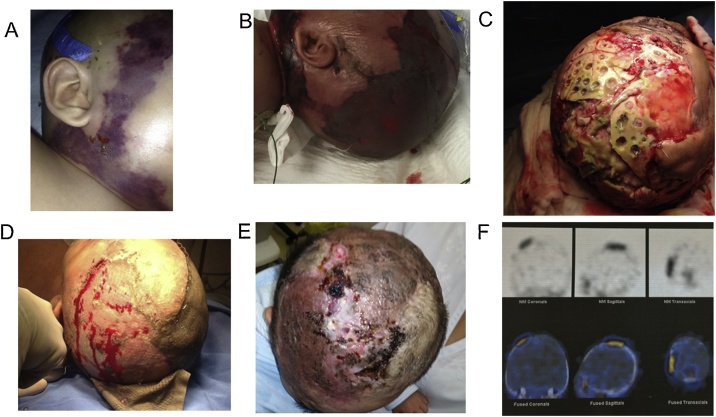
Patient with Crouzon Syndrome, with cranio-cervico-facial involvement after a surgical procedure of cervicofacial advancement whit septic shock and acute and chronic osteomyelitis. A) Bilateral areas of ecchymosis were observed in the cervico-maxillary region. B) Necrotic areas in the cervical, frontal, temporal and occipital regions. C) Infected area with *Pseudomonas aeruginosa*. D) Once the infection was controlled after multiple surgical lavages and debridation, broad spectrum antibiotics, VAC and hyperbaric oxygen the skin grafts were placed. E) The patient was discharged from the hospital. F) Bone scintigraphy.- the bone scan images showed abnormal increased uptake of ^111^In in the right temporal region.

Over the next hours, tissue edema was observed in the cephalic region at the surgical wound. A Computed Tomography scan of the head was performed, an infiltrative soft tissue edema with a probable hemorrhagic component was observed. Antibiotic therapy was adjusted to ceftriaxone and clindamycin due to probable infection of the surgical wound. Two days later, bilateral areas of ecchymosis developed in the cervico-maxillary region. A blister in the right cheek spontaneously ruptured and drained thick yellowish material. Indurated skin and violaceous and well delimited lesions were noticed in some areas with scab formation (Fig. 1A). The antibiotic therapy was changed to meropenem and vancomycin. Despite of the use of broad spectrum intravenous antibiotics his clinical condition worsened. A new clinical examination showed weak pulses, poor skin perfusion and respiratory failure. The patient was placed in mechanical ventilatory support and he was diagnosed with septic shock.

Over the next 48 h, necrotic areas developed in the occipital, frontal, parietal, cervical and upper back regions (Fig. 1B), a new head computed tomography scan of the head showed soft tissue edema and a subgaleal fluid collection with defined borders. The gram stain of the cervical wound revealed gram positive short bacilli suggestive of anaerobes. Surgical lavage and debridation of necrotic tissue up to the muscular plane and some areas of the head were required for the next days, the clinical condition improved, an increase of the platelet count was observed up to 214 × 10^3^/mm^3^ allowing the decrease of ventilatory parameters.

Regardless of the clinical improvement, the patient persisted with fever and hemodynamic instability. An angioresonance showed an inflammatory process in the bilateral frontotemporal occipital areas with epidural, right parietal and periorbital fluid collections, a subdural empyema in the left parietal area, and a suggestive image of temporal artery thrombosis. *Candida albicans* was isolated from a neck tissue sample, *Staphylococcus epidermidis* was isolated from a central venous catheter culture and *Acinetobacter baumannii* was isolated from a head tissue sample in different times. Antibiotic therapy with meropenem and vamcomycin was continued and fluconazole was added to the treatment.

After multiple surgical lavages and debridement of necrotic tissue and skin grafting of the neck and cheeks, the patient showed clinical improvement and was able to extubate.

In order to cover the skull and because of a very large uncovered area, an artificial skin graft was placed in the temporoparietal area, where after few days an infection developed presenting a green secretion under the graft where *Pseudomonas aeruginosa* was isolated, ciprofloxacin was added to the treatment according to the sensitivity pattern (Fig. 1C). The patient presented acute osteomyelitis of the frontoparietal area and surgical lavages and bone debridement was required, the green secretion persisted despite of the treatment. VAC (vacuum assisted closure) therapy and hyperbaric oxygen therapy was added to the management.

The antibiotics, surgical lavages, VAC and hyperbaric oxygen continued until the cranial area was clean and autologous skin grafts from the leg were placed in the skull until the whole skull was covered with tissue and skin (Fig. 1D). After 2 months of intensive care, the patient was discharged from the hospital with oral ciprofloxacin to complete the treatment for cranial osteomyelitis in good general conditions (Fig. 1E).

The patient continued with ambulatory lavages of the head, one month later the patient presented a fistula with yellowish and sometimes green secretion on the right temporal area, a bone scintigraphy with Indium^111^ and Technetium^99^ was performed; the bone scan images showed an abnormal increased uptake of the Indium^111^ in the right temporal region of the skull, confirming chronic osteomyelitis (Fig. 1F). The patient was admitted to the hospital for surgical resection of the affected bone, surgical lavage and skin graft; 2 strains of multi-resistant *Pseudomonas aeruginosa* with different patterns of sensitivity were isolated from the lesion; one was only sensitive to tobramycin which was not available in México and the other one was sensitive to piperacillin/tazobactam. A synergic testing method (the checkerboard) [6] was used to measure in vitro efficacies of various antimicrobial combinations with intermediate sensitivity against the multi-resistant *Pseudomonas aeruginosa*. Ceftazidime and gentamycin were synergistic and together with piperacillin/tazobactam were used to treat the patient during hospitalization. A subclavian catheter was placed and the patient was discharged from the hospital and received ambulatory antibiotic treatment for 6 weeks. One week later after the discharge tobramycin was imported from United States of America and gentamicin was exchanged for tobramycin. The patient fully recovered and is in good condition, there is bone growth in the frontoparietal area.

## 3 Discussion

Pediatric NF is a rare but severe life threatening infection. The process is marked by necrosis of the superficial fascia, neutrophil infiltration of the deep dermis and fascia, thrombosis of the cutaneous microcirculation and the presence of the infectious organism in the necrotic tissue [7].

Although early antibiotic therapy and critical care are necessary in the treatment of patients with NF, early aggressive surgical treatment is crucial for the adequate outcome [3]. Multiple studies describe the need for early and aggressive debridement in NF, however, there are no randomized clinical controlled trial analyzing the timing or extent of surgical debridement [8]. Another adjuvant treatment for NF is the Vacuum-assisted closure and the hyperbaric oxygen therapy.

The Vacuum-assisted closure, is a well-known wound care system for the treatment of complex wounds. The cyclical application of negative pressure can accelerate wound healing, where optimized blood flow increases local oxygenation and promotes angiogenesis, decreases local tissue edema and accelerates removal of excessive fluid from the wound bed, which in turn reduces bacterial contamination [9]. Several authors report the use of Vacuum-Assisted Closure in acute [10] and chronic wounds, the removal of necrotic tissue and development of robust new healthy granulation tissue boosted by obliteration of cavities are the main advantages [11]. In addition, there is decreased bacterial contamination and increased vascularity of the bed. VAC also reduces edema and facilitates wound closure [12,13].

The hyperbaric oxygen therapy has been proposed to improve wound healing and survival in patients with NF by possibly increasing oxygen tension at the ischemic wound bed, facilitating the action of cytotoxic leukocytes, and improving antibiotic delivery via hyperoxygenation [14,15]. There are improvements in tissue oxygenation phagocytosis and edema and impairment of bacterial metabolism and exotoxin production. Furthermore, oxygen has a synergistic effect with antibiotics, enhancing angiogenesis and promoting wound healing [16].

## 4 Conclusion

We describe a case where proper early diagnosis, adequate antibiotic therapy and aggressive surgical debridement of the necrotic tissue combined with VAC and hyperbaric oxygen therapy which is used by many surgeons in the case of serious infections [17] played an important role in the successful treatment. Moreover, special laboratory test methods such as the checkerboard can be used to determine the adequate antimicrobial combination of drugs to obtain a synergistic effect when the patient receives a long period of broad spectrum antibiotics giving rise to multirresistant strains that respond to a combination of antibiotics. A multidisciplinary management and coordination of different pediatric services are fundamental to increase the success in the treatment and survival of this pathology.

## Conflicts of interest

All the authors declare that there is not any conflict of interest regarding the publication of this case report.

## Sources of funding

All the authors declare that there was no study sponsor.

## Ethical approval

The authors declare that the study is exempt from ethical approval from the Hospital Angeles del Pedregal and the Faculty of Medicine, Universidad Nacional Autonoma de México.

## Consent

The written informed consent was obtained from the parents for publication of this case report and accompanying images. A copy of the written consent is available for review by the Editor-in Chief of this journal on request.

## Author’s contribution

RMWC was the infectious diseases specialist, APS and GFC the plastic surgeons, AMB the neurosurgeon and SGM and MLMY the intensive care specialists involved in the multidisciplinary treatment of the patient. AHAA analized the case and with RMWC were major contributors in writing the manuscript. All authors read and approved the final manuscript.

## Registration of research studies

Not applicable.

## Guarantor

Rosa Maria Wong Chew.

## Provenance and review

Not commissioned, reviewed by Editor.
